# A deafness-associated tRNA^His^ mutation alters the mitochondrial function, ROS production and membrane potential

**DOI:** 10.1093/nar/gku466

**Published:** 2014-06-11

**Authors:** Shasha Gong, Yanyan Peng, Pingping Jiang, Meng Wang, Mingjie Fan, Xinjian Wang, Hong Zhou, Huawei Li, Qingfeng Yan, Taosheng Huang, Min-Xin Guan

**Affiliations:** 1 Institute of Genetics, Zhejiang University, Hangzhou, Zhejiang, China 310058; 2 Division of Human Genetics, Cincinnati Children's Hospital Medical Center, Cincinnati, OH, USA 45229; 3 Department of Otology and Skull Base Surgery, Eye and ENT Hospital, Fudan University, Shanghai, China 200031

## Abstract

In this report, we investigated the molecular genetic mechanism underlying the deafness-associated mitochondrial tRNA^His^ 12201T>C mutation. The destabilization of a highly conserved base-pairing (5A-68U) by the m.12201T>C mutation alters structure and function of tRNA^His^. Using cybrids constructed by transferring mitochondria from lymphoblastoid cell lines derived from a Chinese family into mtDNA-less (ρ^o^) cells, we showed ∼70% decrease in the steady-state level of tRNA^His^ in mutant cybrids, compared with control cybrids. The mutation changed the conformation of tRNA^His^, as suggested by slower electrophoretic mobility of mutated tRNA with respect to the wild-type molecule. However, ∼60% increase in aminoacylated level of tRNA^His^ was observed in mutant cells. The failure in tRNA^His^ metabolism was responsible for the variable reductions in seven mtDNA-encoded polypeptides in mutant cells, ranging from 37 to 81%, with the average of ∼46% reduction, as compared with those of control cells. The impaired mitochondrial translation caused defects in respiratory capacity in mutant cells. Furthermore, marked decreases in the levels of mitochondrial ATP and membrane potential were observed in mutant cells. These mitochondrial dysfunctions caused an increase in the production of reactive oxygen species in the mutant cells. The data provide the evidence for a mitochondrial tRNA^His^ mutation leading to deafness.

## INTRODUCTION

Deafness is one of the major public health problems, affecting 360 million persons worldwide. Deafness can be grouped into syndromic deafness (hearing loss with other medical problems such as diabetes), and non-syndromic deafness (hearing loss is the only obvious medical problem). Mutations in mitochondrial DNA (mtDNA) are one of the important causes of syndromic and non-syndromic deafness (1–3). In particular, the 1555A>G and 1494C>T mutations in the 12S rRNA gene have been associated with aminoglycoside-induced and non-syndromic deafness in many families worldwide (3–5). Mitochondrial tRNA genes are another hot spots for mutations associated with both syndromic and non-syndromic deafness (6,7). The most prevalent mtDNA mutation associated with syndromic deafness was the m.3243A>G mutation in the tRNA^Leu(UUR)^ gene (8). The non-syndromic deafness-associated tRNA mutations were the tRNA^Ser(UCN)^ 7445A>G, 7472insC, 7505T>C, 7510T>C and 7511T>C, tRNA^His^ 12201T>C and tRNA^Ile^ 4295A>G mutations (2,9–14). These mutations have structural and functional consequences, including the processing of RNA precursors, nucleotide modification and aminoacylation (6,15). The m.7445A>G mutation altered the processing of the tRNA^Ser(UCN)^ precursor (16), while the m.4295A>G mutation may affect the nucleotide modification at position 37, 3′ end adjacent to anticodon of the tRNA^Ile^ (17). Furthermore, the m.7510T>C and m.7511T>C mutations disrupted the Watson–Crick base-pairing(s) at acceptor stem of tRNA^Ser(UCN)^, thereby altering the tRNA metabolisms (11,18).

**Figure 1.**
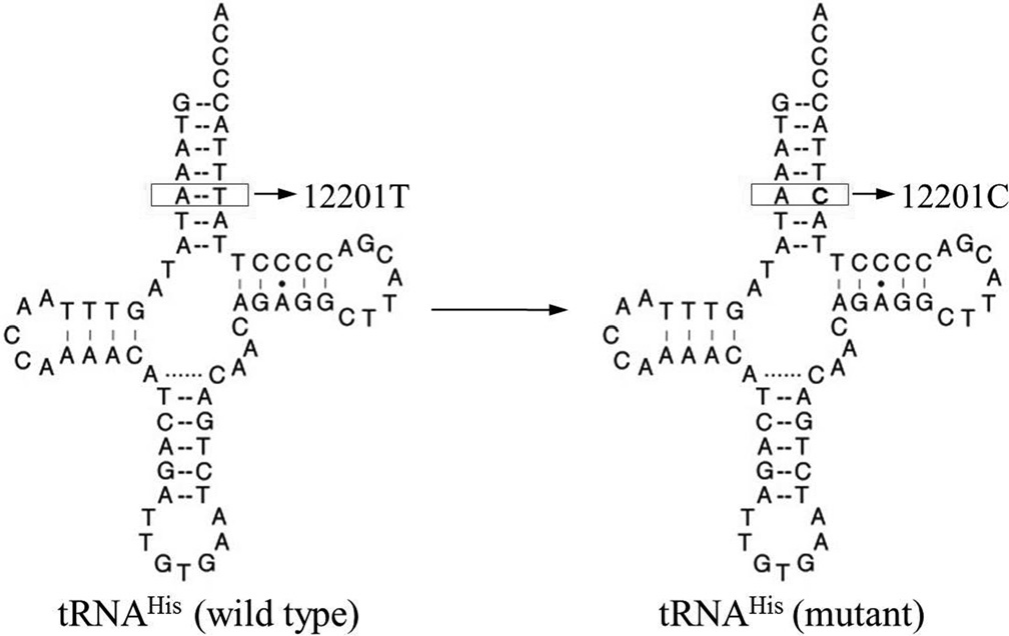
Cloverleaf structure of human mitochondrial tRNA^His^. An arrow denotes the location of the m.12201T>C mutation.

The m.12201T>C mutation in the tRNA^His^ gene was associated with maternally transmitted non-syndromic deafness in a large Han Chinese pedigree (14). As shown in Figure 1, the m.12201T>C mutation is localized at a highly conserved nucleotide (U68), which forms a base-pairing (5A-68U) on the acceptor stem of the tRNA^His^ (14). It was hypothesized that the destabilization of the base-pairing (5A-68U) by the m.12201T>C mutation altered the structure and function of tRNA^His^. In particular, the mutation may affect the aminoacylation capacity and stability of this tRNA. A failure in tRNA metabolism leads to the impairment of mitochondrial translation and respiration (14). It was also proposed that mitochondrial dysfunctions caused by the tRNA mutation alter the mitochondrial membrane potential, production of ATP and reactive oxygen species (ROS). To further investigate the pathogenic mechanism of the m.12201T>C mutation in the Chinese family, cybrid cell lines were constructed by transferring mitochondria from lymphoblastoid cell lines derived from an affected matrilineal relative carrying the mtDNA mutation and from a control individual lacking the mtDNA mutation, into human mtDNA-less (ρ°) cells (19,20). These cybrid cell lines were first examined for the presence and degree of the mtDNA mutation. These cell lines were then assessed for the effects of the mtDNA mutation on the tRNA metabolism, mitochondrial translation, respiration, production of ATP and ROS, as well as mitochondrial membrane potential.

## MATERIALS AND METHODS

### Cell lines and culture conditions

Immortalized lymphoblastoid cell lines derived from one affected matrilineal relative (IV-11) of the Chinese family carrying the m.12201T>C mutation and one genetically unrelated Chinese control individual belonging to the same mtDNA haplogroup Z3 but lacking the mutation (H7) (Supplemental Table S1) were grown in RPMI 1640 medium with 10% fetal bovine serum (FBS). The bromodeoxyuridine (BrdU) resistant 143B.TK^−^ cell line was grown in Dulbecco's Modified Eagle Medium (DMEM) (Life Technologies) (containing 4.5 mg of glucose and 0.11 mg pyruvate/ml), supplemented with 100 μg of BrdU/ml and 5% FBS. The mtDNA-less ρ°206 cell line, derived from 143B.TK^−^ (20) was grown under the same conditions as the parental line, except for the addition of 50 μg of uridine/ml. All cybrid cell lines constructed with enucleated lymphoblastoid cell lines were maintained in the same medium as the 143B.TK^−^ cell line.

### Mitochondria mediated ρ°206 cell transformation

Immortalized lymphoblastoid cell lines derived from one affected member of the Chinese family (IV-11) and one Chinese control individual (H7) were used for the generation of cybrid cell lines. Transformation by cytoplasts of mtDNA less ρ°206 cells was performed as described elsewhere (19–21).

### Mitochondrial DNA analysis

An analysis for the presence and level of the m.12201T>C mutation in the tRNA^His^ gene was carried out as described in the supplemental materials. The quantification of mtDNA copy numbers from different cybrids was performed by slot blot hybridization as detailed elsewhere (22).

### Mitochondrial tRNA analysis

Total mitochondrial RNA were obtained using TOTALLY RNA™ kit (Ambion) from mitochondria isolated from the cybrid cell lines (∼4.0 × 10^8^ cells), as described previously (23). Two micrograms of total mitochondrial RNA were electrophoresed through a 10% polyacrylamide/7 M urea gel in Tris–borate–Ethylenediaminetetraacetic acid (EDTA) buffer (TBE) (after heating the sample at 65°C for 10 min), and then electroblotted onto a positively charged nylon membrane (Roche) for the hybridization analysis with oligodeoxynucleotide probes. Oligodeoxynucleosides used for digoxigenin (DIG) labeled probes of tRNA^His^, tRNA^Thr^, tRNA^Lys^, tRNA^Leu(CUN)^, tRNA^Gly^ and 5S RNA were described as elsewhere (14,24,25). DIG-labeled oligodeoxynucleotides were generated by using DIG oligonucleotide Tailing kit (Roche). The hybridization was carried out as detailed elsewhere (14,24,25). Quantification of density in each band was made as detailed previously (14,25).

### Mitochondrial tRNA aminoacylation analysis

Total mitochondrial RNAs were isolated under acid conditions. Two micrograms of total mitochondrial RNAs was electrophoresed at 4°C through an acid (pH 5.2) 10% polyacrylamide–7 M urea gel to separate the charged and uncharged tRNA as detailed elsewhere (25,26). The gels were then electroblotted onto a positively charged nylon membrane (Roche) for the hybridization analysis with oligodeoxynucleotide probes as described above. Quantification of density in each band was performed as detailed previously (26).

### Western blot analysis

Twenty micrograms of proteins obtained from lysed cells were denatured and loaded on sodium dodecyl sulfate polyacrylamide gels. Afterward, the proteins were transferred to polyvinylidene difluoride (PVDF) membrane and subjected to Western blotting. Membranes were blocked in Tris-Buffered Saline and Tween20 (TBST) (150 mM NaCl, 10 mM Tris–HCl, pH 7.5 and 0.1% Tween 20) containing 5% (w/v) milk, then incubated with the corresponding primary and secondary antibodies. The primary antibodies used for this experiment were the rabbit anti-ND1, ND5 and A6, mouse anti-CO_1_ and CO_2_ (Abcam), rabbit anti-ND4 and CYTB (Santa Cruz), and mouse anti-actin (Beyotime). Peroxidase AffiniPure goat anti-mouse IgG and goat anti-rabbit IgG (Jackson) were used as a secondary antibody and protein signals were detected using the ECL system (CWBIO).

### Measurements of oxygen consumption

The rates of oxygen consumption in cybrid cell lines were measured with a Seahorse Bioscience XF-96 extracellular flux analyzer (Seahorse Bioscience), as detailed elsewhere (27,28). XF96 creates a transient, 7 μl-chamber in specialized microplates that allows for the determination of oxygen and proton concentrations in real time. To allow comparison between different experiments, data are expressed as the rate of oxygen consumption in pmol/min or the rate of extracellular acidification in mpH/min, normalized to cell protein in individual wells determined by the Bradford protein assay (Bio-Rad). A density of 20 000 cells per well in 96-well plate was coated with Cell-Tak™ adhesive. The rates of O_2_ were determined under basal condition and the addition of oligomycin (1.5 μM), carbonyl cyanide *p*-(trifluoromethoxy) phenylhydrazone (FCCP) (0.5 μM), rotenone (1 μM) and antimycin A (1 μM), as detailed elsewhere (27,28).

### ATP measurements

The CellTiter-Glo^®^ Luminescent Cell Viability Assay kit (Promega) was used for ATP assay according to the manufacturer's instructions (29). Briefly, the assay buffer and substrate were equilibrated to room temperature, and the buffer was transferred to and gently mixed with the substrate to obtain a homogeneous solution. After a 30 min equilibration of the cell plate to room temperature, 100 μl of the assay reagent was added into each well with 20 000 cells and the content was mixed for 2 min on an orbital shaker to induce cell lysis. After 10 min incubation in room temperature, the luminescence was read on a microplate reader (Syneregy H1, Bio-Tek).

### Assessment of mitochondrial membrane potential

Mitochondrial membrane potential was assessed with JC-10 Assay Kit-Microplate (Abcam) following general manufacturer's recommendations with some modifications (30,31). In brief, cells were plated onto 96-well cell culture plate overnight in growth medium. JC-10 dye-loading solution was added for 30 min at 37°C, 5% CO_2_. Alternatively, plated cells were preincubated with 10 μM of the protonophore uncoupler carbonyl cyanide 3-chlorophenylhydrazone (CCCP) for 30 min at 37°C, 5% CO_2_ prior to staining with JC-10 dye. The fluorescent intensities for both J-aggregates and monomeric forms of JC-10 were measured at Ex/Em = 490/530 and 490/590 nm with a microplate reader (Syneregy H1, Bio-Tek).

### ROS measurements

ROS measurements were performed following the procedures detailed elsewhere (29,32). Briefly, approximate 2 × 10^6^ cells of each cell line were harvested, resuspended in PBS supplemented with 100 μM of 2′,7′-dichlorodihydrofluorescein diacetate (DCFH-DA) and then incubated at 37°C for 20 min. After washing with PBS twice, cells were resuspended in PBS in the presence of 2 mM freshly prepared H_2_O_2_ and 2% FBS and then incubated at room temperature for another 45 min. Cells were further washed with PBS and resuspended with 1 ml of PBS with 0.5% paraformaldehyde. Samples with or without H_2_O_2_ stimulation were analyzed by BD-LSR II flow cytometer system (Beckton Dickson, Inc.), with an excitation at 488 nm and emission at 529 nm. Ten thousand events were analyzed in each sample.

### Statistical analysis

Statistical analysis was carried out using the Student's unpaired, two-tailed *t*-test contained in the Microsoft-Excel program. Unless indicated otherwise, a *P*-value <0.05 was considered statistically significant.

## RESULTS

### The construction of cybrid cell lines

The lymphoblastoid cells derived from one affected subject (IV-11) and one control individual (H7) were enucleated, and subsequently fused to a large excess of mtDNA-less human ρ°206 cells, derived from the 143B.TK^−^ cell line (19). The cybrid clones were isolated by growing the fusion mixtures in selective DMEM medium, containing BrdU and lacking uridine (19,20). Between 25 and 45 days after fusion, 10–15 presumptive mitochondrial cybrids derived from each donor cell lines were isolated, and subsequently analyzed for the presence and level of the m.12201T>C mutation (14). The results confirmed the absence of the mtDNA mutation in the control clones and its presence in homoplasmy in all cybrids derived from the mutant cell line (Supplemental figure). Three cybrids derived from each donor cell line with similar mtDNA copy numbers were used for the biochemical characterization described below.

### Marked decrease in the levels of tRNA^His^

**Figure 2.**
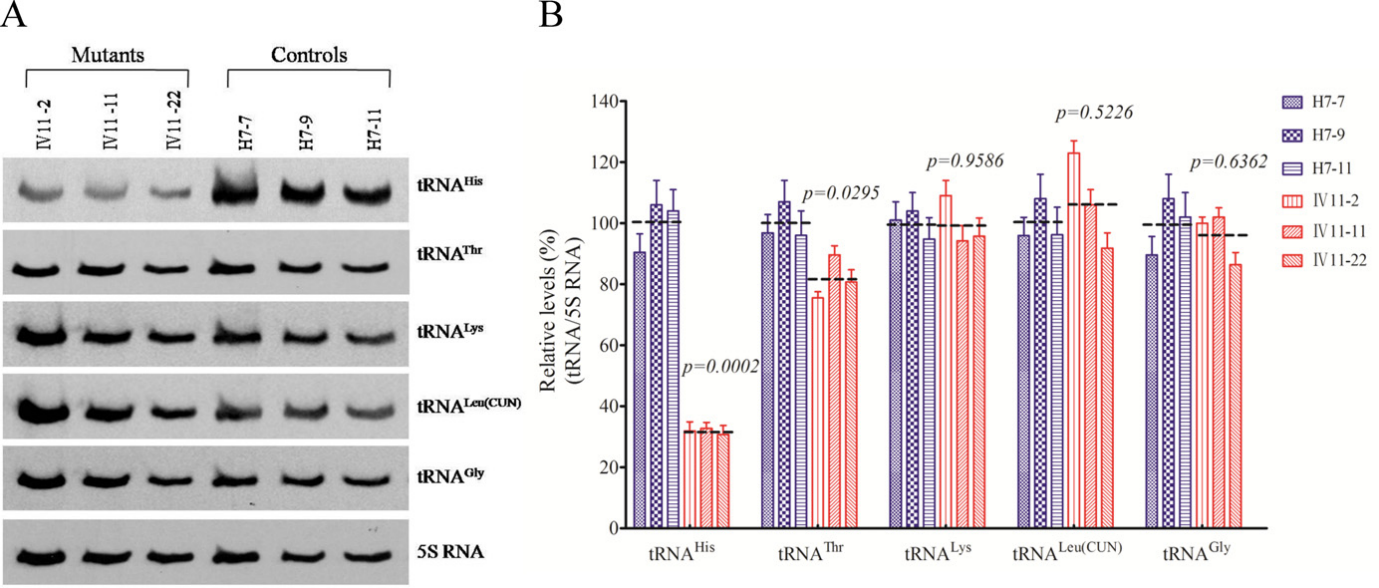
Northern blot analysis of mitochondrial tRNA. (**A**) Two micrograms of total mitochondrial RNA from various cell lines were electrophoresed through a denaturing polyacrylamide gel, electroblotted and hybridized with DIG-labeled oligonucleotide probes for the tRNA^His^, tRNA^Thr^, tRNA^Lys^, tRNA^Leu(CUN)^, tRNA^Gly^ and 5S RNA, respectively. (**B**) Quantification of mitochondrial tRNA levels. Average relative tRNA content per cell, normalized to the average content per cell of 5S RNA in three cybrid cell lines derived from one affected subject (IV-11) carrying the m.12201T>C mutation and three cybrid cell lines derived from one Chinese control subject (H7). The values for the latter are expressed as percentages of the average values for the control cell lines. The calculations were based on three independent determinations of each tRNA content in each cell line and three determinations of the content of 5S RNA in each cell line. The error bars indicate two standard errors of the means. *P* indicates the significance, according to the *t*-test, of the differences between mutant and control cell lines.

To further examine whether the m.12201T>C mutation affects the stability of tRNA^His^, we subjected mitochondrial RNAs from cybrids to northern blots and hybridized them with DIG-labeled oligodeoxynucleotide probes for tRNA^His^, tRNA^Thr^, tRNA^Lys^, tRNA^Leu(CUN)^ and tRNA^Gly^ as well as a nucleus-encoded mitochondrial 5S RNA. As shown in Figure 2A, the amount of tRNA^His^ in three mutant cell lines were markedly decreased, compared with those in three control cybrid cell lines. For comparison, the average level of each tRNA in control or mutant cell lines was normalized to the average levels in the same cell line for reference 5S RNA. As shown in Figure 2B, the average steady-state levels of tRNA^His^ in three cell lines were 31.8% (*P* = 0.0002) of three controls cell lines after normalization to 5S RNA. However, the average steady-state levels of tRNA^Thr^, tRNA^Lys^, tRNA^Leu(CUN)^ and tRNA^Gly^ in three mutant cell lines were 84.2, 99.7, 109.7 and 96.3%, respectively of those in three cell lines after normalization to 5S RNA.

### Altered aminoacylation of tRNA^His^

**Figure 3.**
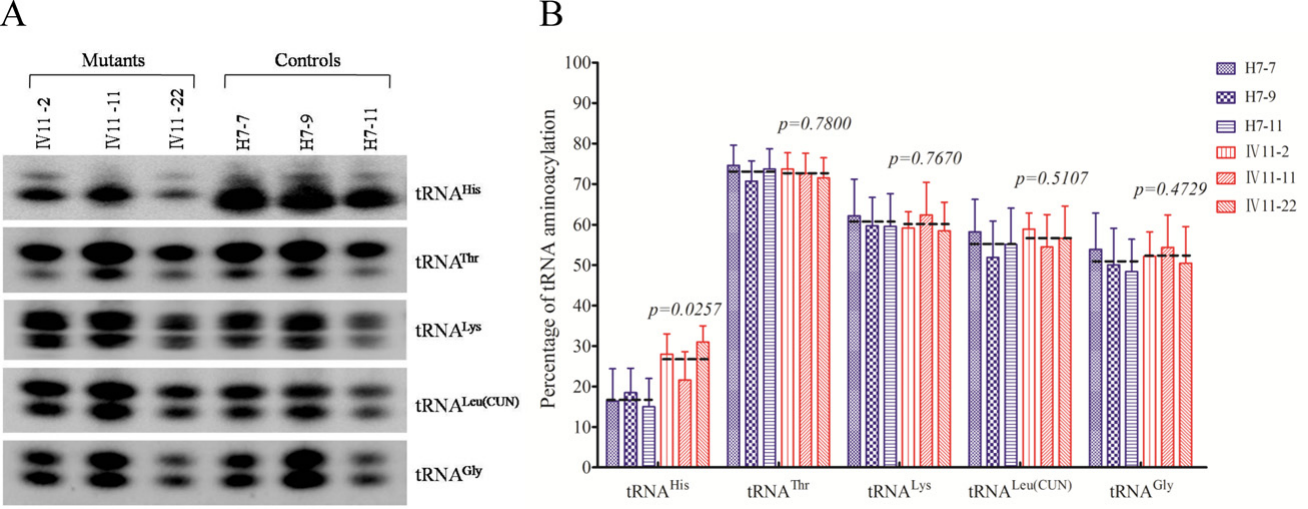
*In vivo* aminoacylation assays. (**A**) two micrograms of total mitochondrial RNA purified from six cell lines (the same as in Figure 2A) under acid conditions were electrophoresed at 4°C through an acid (pH 5.2) 10% polyacrylamide–7 M urea gel, electroblotted, and hybridized with a DIG-labeled oligonucleotide probe-specific for the tRNA^His^. The blots were then stripped and rehybridized with tRNA^Thr^, tRNA^Lys^, tRNA^Leu(CUN)^ and tRNA^Gly^, respectively. (**B**) *In vivo* aminoacylated proportions of tRNA^His^, tRNA^Thr^, tRNA^Lys^, tRNA^Leu(CUN)^ and tRNA^Gly^ in the mutant and controls. The calculations were based on three independent determinations. Graph details and symbols are explained in the legend to Figure 2.

The aminoacylation capacities of tRNA^His^, tRNA^Thr^, tRNA^Lys^, tRNA^Leu(CUN)^ and tRNA^Gly^ in control and mutant cell lines were examined by the use of electrophoresis in an acid polyacrylamide/urea gel system to separate uncharged tRNA species from the corresponding charged tRNA, electroblotting and hybridizing with above tRNA probes (26). As shown in Figure 3, the upper band represented the charged tRNA, and the lower band was uncharged tRNA. Electrophoretic patterns showed that either charged or uncharged tRNA^His^ in cell lines carrying the m.12201T>C mutation migrated slower than those of cell lines lacking this mutation. However, there were no obvious differences in electrophoretic mobility of tRNA^Thr^, tRNA^Lys^, tRNA^Leu(CUN)^ and tRNA^Gly^ between the cell lines carrying the m.12201T>C mutation and cell lines lacking this mutation. Notably, the efficiencies of aminoacylated tRNA^His^ in these mutant cell lines reflected 62% increase, ranged from 30 to 86%, relative to the average control values (*P* = 0.0257). However, the levels of aminoacylation in tRNA^Thr^, tRNA^Lys^, tRNA^Leu(CUN)^ and tRNA^Gly^ in mutant cell lines were comparable with those in the control cell lines.

### Reduction in the level of mitochondrial proteins

**Figure 4.**
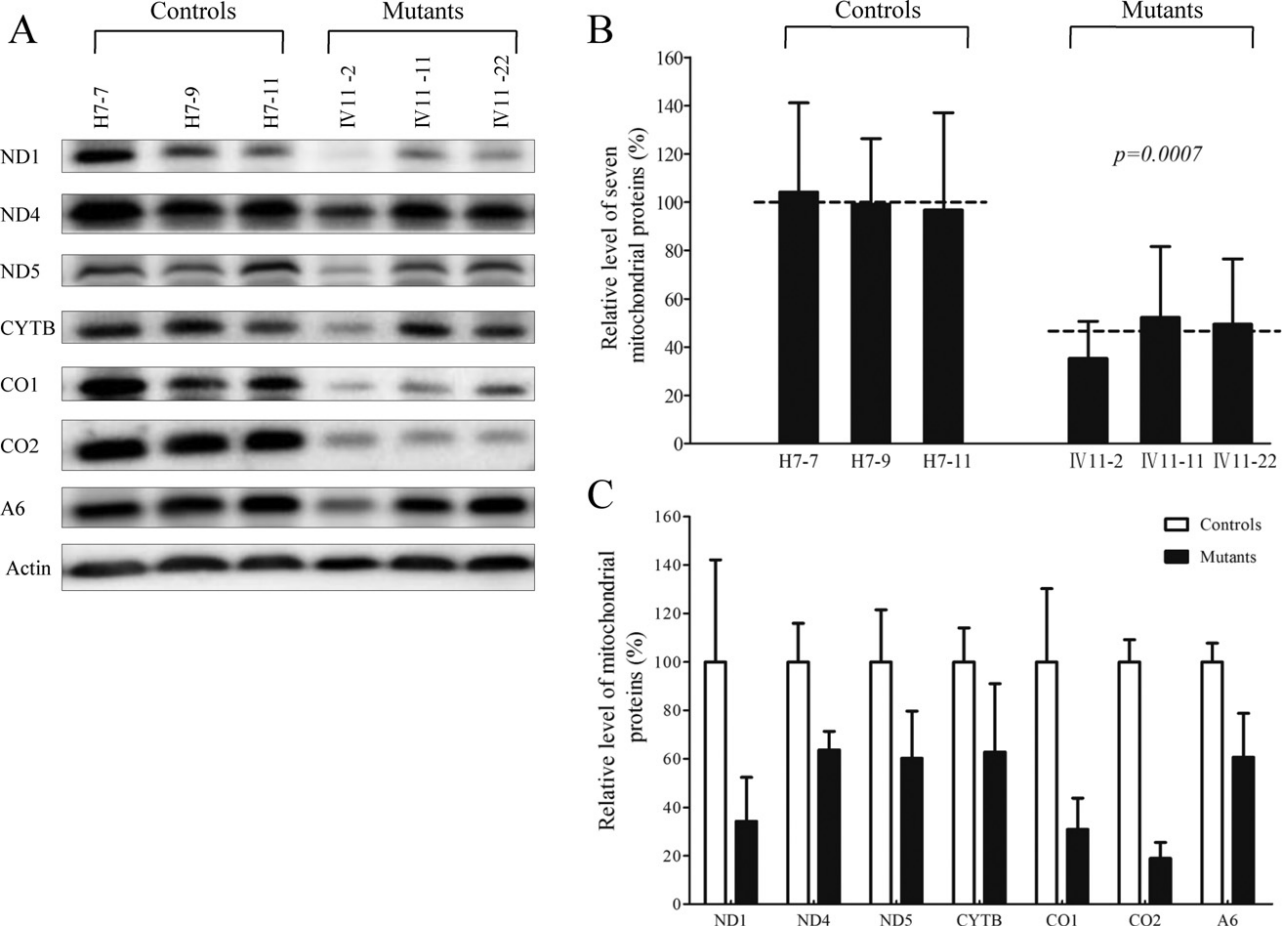
Western blot analysis of mitochondrial proteins. (**A**) Twenty micrograms of total cellular proteins from various cell lines were electrophoresed through a denaturing polyacrylamide gel, electroblotted and hybridized with seven respiratory complex subunits in mutant and control cells with β-actin as a loading control. p.MT-COI and p.MT-COII, indicate subunits I and II of cytochrome *c* oxidase; p.MT-ND1, p.MT-ND4 and p.MT-ND5, subunits 1, 4 and 5 of the reduced nicotinamide–adenine dinucleotide dehydrogenase; p.MT-A6, subunit 6 of the H^+^-ATPase; and p.MT-CYTB, apocytochrome *b*. (**B**) Quantification of mitochondrial protein levels. Average relative p.MT-COI, p.MT-COII, p.MT-ND1, p.MT-ND4, p.MT-ND5, p.MT-A6 and p.MT-CYTB content per cell, normalized to the average content per cell of β-actin in three mutant cell lines carrying the m.12201T>C mutation and three control cell lines lacking the mutation. (C) Quantification of 7 respiratory complex subunits. Average relative p.MT-COI, p.MT-COII, p.MT-ND1, p.MT-ND4, p.MT-ND5, p.MT-A6 and p.MT-CYTB content per cell, normalized to the average content per cell of β-actin in three mutant cell lines carrying the m.12201T>C mutation and three control cell lines lacking the mutation. The values for the mutant cell lines are expressed as percentages of the average values for the control cell lines. The calculations were based on three independent determinations. Graph details and symbols are explained in the legend to figure 2. The values for the latter are expressed as percentages of the average values for the control cell line. The calculations were based on three independent determinations. Graph details and symbols are explained in the legend to Figure 2.

To further determine whether the impairment of mitochondrial translation occurred in the cell lines carrying the m.12201T>C mutation, a Western blot analysis was carried out to examine the steady state levels of seven respiratory complex subunits in mutant and control cells with β-actin as a loading control. As shown in Figure 4, the levels of p.MT-CO1 and p.MT-CO2, subunits I and II of cytochrome *c* oxidase; p.MT-ND1, p.MT-ND4 and p.MT-ND5, subunits 1, 4 and 5 of NADH dehydrogenase; p.MT-A6, subunit 6 of the H^+^-ATPase; p.MT-CYTB, apocytochrome *b* were decreased in three mutant cell lines, as compared with those of three control cell lines. As shown in Figure 4B, the overall levels of seven mitochondrial translation products in the mutant cell lines was decreased relative to the mean value measured in the control cell lines by ∼35–52%, with an average of 46% (*P* = 0.0007). Notably, the average levels of p.MT-ND1, p.MT-ND4, p.MT-ND5, p.MT-CO1, p.MT-CO2, p.MT-A6 and p.MT-CYTB in the mutant cells were 34, 64, 60, 43, 19, 61 and 63% of the average values of control cells, respectively. However, the levels of synthesis of polypeptides in mutants relative to that in controls did not correlate with either the number of codons or proportion of histidine residues (Supplemental Table S2). This result was in contrast to previous studies with the deafness-associated m.7445A>G mutation in the precursor of tRNA^Ser(UCN)^ gene (16).

### Respiration defects

**Figure 5.**
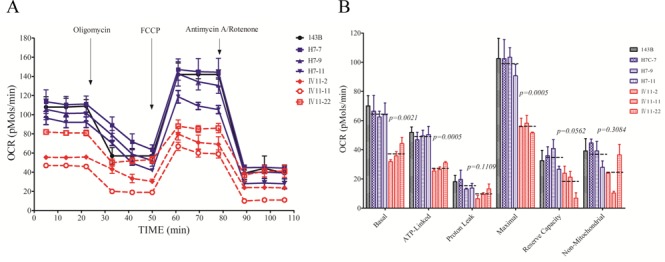
Respiration assays. (**A**) An analysis of O_2_ consumption in the various cell lines using different inhibitors. The rates of O_2_ (OCR) were first measured on 2 × 10^4^ cells of each cell line under basal condition and then sequentially added to oligomycin (1.5 μM), carbonyl cyanide *p*-(trifluoromethoxy) phenylhydrazone (FCCP) (0.5 μM), rotenone (1 μM) and antimycin A (1 μM) at indicated times to determine different parameters of mitochondrial functions. (**B**) Graphs presented the ATP-linked OCR, proton leak OCR, maximal OCR, reserve capacity and non-mitochondrial OCR in mutant and control cell lines. Non-mitochondrial OCR was determined as the OCR after rotenone/antimycin A treatment. Basal OCR was determined as OCR before oligomycin minus OCR after rotenone/antimycin A. ATP-linked OCR was determined as OCR before oligomycin minus OCR after oligomycin. Proton leak was determined as basal OCR minus ATP-linked OCR. Maximal was determined as the OCR after FCCP minus non-mitochondrial OCR. Reserve capacity was defined as the difference between maximal OCR after FCCP minus basal OCR. The average of four determinations for each cell line is shown, the horizontal dashed lines represent the average value for each group. Graph details and symbols are explained in the legend to Figure 2.

To evaluate if the m.12201T>C mutation alters cellular bioenergetics, we examined the oxygen consumption rates (OCR) of cell lines derived from three mutant cell lines carrying the m.12201T>C mutation and three control cell lines. As shown in Figure 5, the basal OCR in the mutant cell lines was ∼58% (*P* = 0.0021) relative to the mean value measured in the control cell lines. To investigate which of the enzyme complexes of the respiratory chain was affected in the mutant cell lines, OCR were measured after the sequential addition of oligomycin (inhibit the ATP synthase), FCCP (to uncouple the mitochondrial inner membrane and allow for maximum electron flux through the ETC), rotenone (to inhibit complex I) and antimycin A (to inhibit complex III). The difference between the basal OCR and the drug-insensitive OCR yields the amount of ATP-linked OCR, proton leak OCR, maximal OCR, reserve capacity and non-mitochondrial OCR. As shown in Figure 5, the ATP-linked OCR, proton leak OCR, maximal OCR, reserve capacity and non-mitochondrial OCR in mutant cell lines were ∼56, 63, 57, 53 and 68%, relative to the mean value measured in the control cell lines (*P* = 0.0005, 0.1109, 0.0005, 0.0562 and 0.3084), respectively.

### Reduced level in mitochondrial ATP production

**Figure 6.**
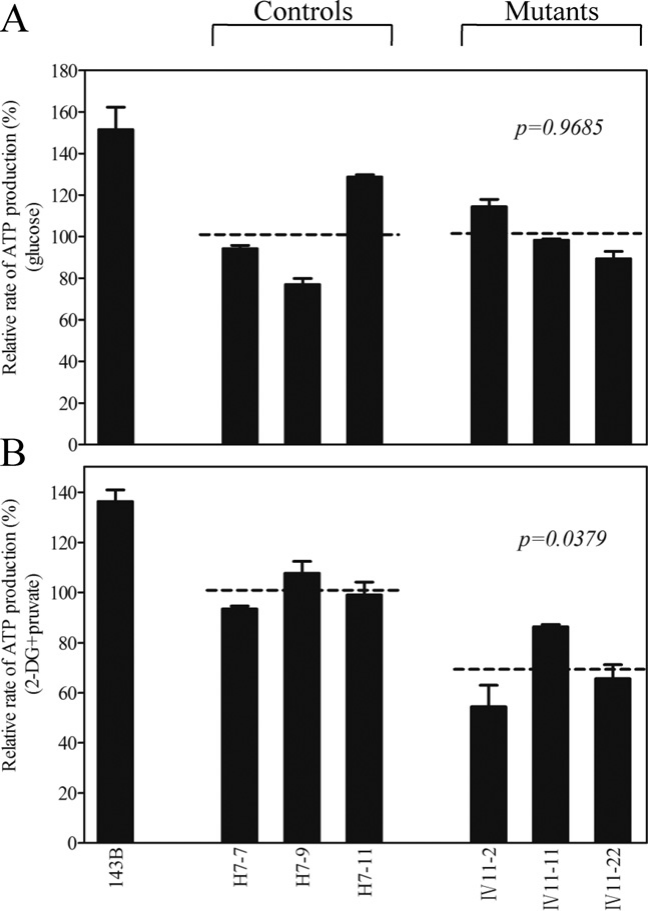
Measurement of cellular and mitochondrial ATP levels using bioluminescence assay. Cells were incubated with 10 mM glucose or 5 mM 2-deoxy-d-glucose plus 5 mM pyruvate to determine ATP generation under mitochondrial ATP synthesis. Average rates of ATP level per cell line and are shown: (**A**) ATP level in total cells; (**B**) ATP level in mitochondria. Six to seven determinations were made for each cell line. Graph details and symbols are explained in the legend to Figure 2.

The capacity of oxidative phosphorylation in mutant and wild-type cells was examined by measuring the levels of cellular and mitochondrial ATP using a luciferin/luciferase assay. Populations of cells were incubated in the media in the presence of glucose, and 2-deoxy-d-glucose with pyruvate (29). As shown in Figure 6A, the levels of ATP production in mutant cells in the presence of glucose (total cellular levels of ATP) were comparable with those measured in the control cell lines. By contrast, as shown in Figure 6B, the levels of ATP production in mutant cell lines, in the presence of pyruvate and 2-deoxy-d-glucose to inhibit the glycolysis (mitochondrial levels of ATP), ranged from 54 to 86%, with an average of 69% relative to the mean value measured in the control cell lines (*P* = 0.0379).

### Decrease in mitochondrial membrane potential

**Figure 7.**
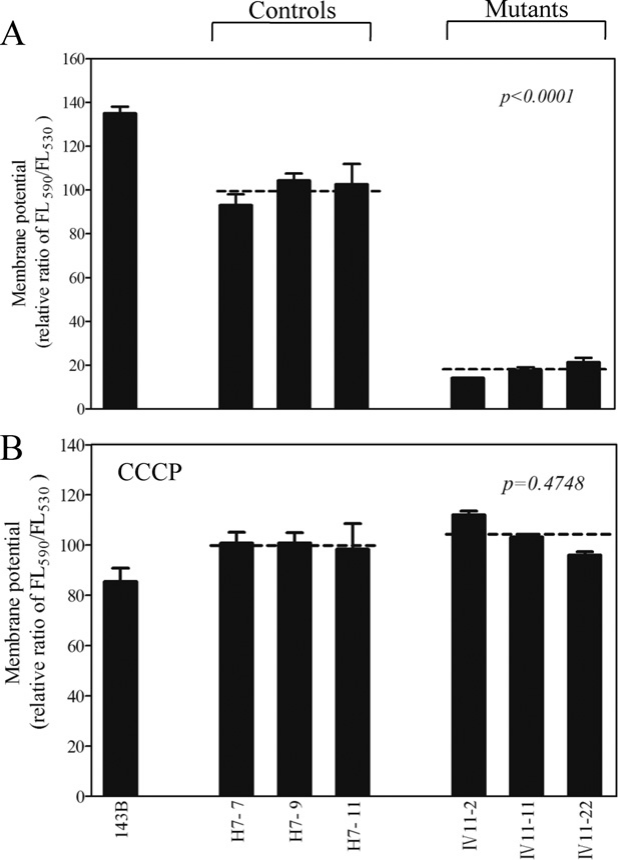
Mitochondrial membrane potential analysis. The mitochondrial membrane potential (Δ*Ψ*_m_) was measured in three mutant and three control cell lines using a fluorescence probe JC-10 assay system. The ratio of fluorescence intensities Ex/Em = 490/590 and 490/530 nm (FL_590_/FL_530_) were recorded to delineate the Δ*Ψ*_m_ level of each sample. The relative ratios of FL_590_/FL_530_ geometric mean between mutant and control cell lines were calculated to reflect the level of Δ*Ψ*_m_. Relative ratio of JC-10 fluorescence intensities at Ex/Em = 490/530 and 490/590 nm in absence (**A**) and presence (**B**) of 10 μM of carbonyl cyanide 3-chlorophenylhydrazone (CCCP). The average of three to five determinations for each cell line is shown. Graph details and symbols are explained in the legend to Figure 2.

The mitochondrial membrane potential (Δ*Ψ*_m_) changes were measured in three mutant and three control cell lines using a fluorescence probe JC-10 assay system. The ratio of fluorescence intensities Ex/Em = 490/590 and 490/530 nm (FL_590_/FL_530_) were recorded to delineate the Δ*Ψ*_m_ level of each sample. The relative ratios of FL_590_/FL_530_ geometric mean between mutant and control cell lines were calculated to represent the level of Δ*Ψ*_m_ As shown in Figure 7, the levels of the Δ*Ψ*_m_ in the mutant cell lines carrying m.12201T>C mutation ranged from 14.2 and 21.4%, with an average 17.7% (*P* < 0.0001) of the mean value measured in the control cell lines. In contrast, the levels of Δ*Ψ*_m_ in mutant cells in the presence of CCCP were comparable with those measured in the control cell lines (*P* = 0.4748).

### The increase of ROS production

**Figure 8.**
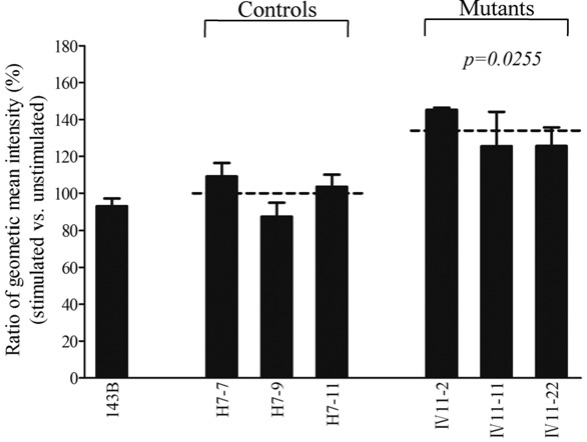
Ratio of geometric mean intensity between levels of the ROS generation in the vital cells with or without H_2_O_2_ stimulation. The rates of production in ROS from three mutant cell lines and three control cell lines were analyzed by BD-LSR II flow cytometer system with or without H_2_O_2_ stimulation. The relative ratio of intensity (stimulated versus unstimulated with H_2_O_2_) was calculated. The average of three determinations for each cell line is shown. Graph details and symbols are explained in the legend to Figure 2.

The levels of the ROS generation in the vital cells derived from three mutant cell lines carrying the m.12201T>C mutation and three control cell lines lacking the mutation were measured with flow cytometry under normal and H_2_O_2_ stimulation (29,32). Geometric mean intensity was recorded to measure the rate of ROS of each sample. The ratio of geometric mean intensity between unstimulated and stimulated with H_2_O_2_ in each cell line was calculated to delineate the reaction upon increasing level of ROS under oxidative stress. As shown in Figure 8, the levels of ROS generation in the mutant cell lines carrying the m.12201T>C mutation ranged from 126 and 145%, with an average 132% (*P* = 0.0255) of the mean value measured in the control cell lines.

## DISCUSSION

In the present study, we investigated the pathogenetic mechanism of the deafness-associated m.12201T>C mutation in the tRNA^His^ gene. The mutation is localized at a highly conserved nucleotide (68U) to form a base-pairing (5A-68U) on the acceptor stem of the tRNA^His^ (33). This nucleotide may act as a discriminator responsible for the stability and identity of tRNA (33,34). The 5A-68U base-pairing may also play an important role in the recognition by its cognate aminoacyl-tRNA synthetase (35). It was hypothesized that the anticipated destabilization of base-pairing (5A-68U) by the m.12201T>C mutation leads to the structural and functional alterations in this tRNA. The primary defect in this mutation appeared to alter the stability and aminoacylation of mutant tRNA^His^. In fact, the m.12201T>C mutation changed the conformation of tRNA^His^, as suggested by slower electrophoretic mobility of mutated tRNA with respect to the wild-type molecule, as in the case of the tRNA^Thr^ 15927G>A mutation (24). However, the aminoacylation level of the tRNA^His^ but no other tRNAs was increased in mutant cell lines, as compared with controls. The increased levels of aminoacylated tRNA^His^ in mutant cell lines may be due to the instability of the mutant tRNA, where aminoacylation may provide some level of stabilization by compensatory effect (36,37). Alternatively, the mutant tRNA^His^ improperly charged by cognate amino acid(s) may contribute to the increasing aminoacylation level of tRNA^His^ (38). A failure to aminoacylate tRNA properly then makes the mutant tRNA^His^ to be metabolically less stable and more subject to degradation, thereby lowering the level of the tRNA, as in the case of 3243A>G mutation in the tRNA^Leu(UUR)^ (25,39). In the present study, 70% reduction in the steady-state level of tRNA^His^ observed in cybrids was consistent with the previous observation in lymphoblastoid cell lines carrying the m.12201T>C mutation (14). These results strongly support the notion that the reduced level of tRNA^His^ in mutant cells is indeed below the proposed threshold to produce a clinical phenotype associated with a mitochondrial tRNA mutation (16,18,39). In this investigation, it is interesting that 16% reduction in the steady-state level of tRNA^Thr^ was also observed in the cybrid cell lines carrying the m.12201T>C mutation. It is likely that the altered tRNA^His^ stability and aminoacylation may mediate the tRNA metabolism, thereby reducing the level of tRNA^Thr^ (25).

A shortage or improper aminoacylation of the tRNA^His^ then leads to the impairment of mitochondrial protein synthesis. In the present study, a markedly decreased level of mitochondrial proteins (an average decrease of ∼46%) was observed in mutant cybrid cell lines, as compared to the average levels in control cell lines. The reduced level of variable mitochondrial proteins detected in cybrids using a Western blot analysis was comparable with the reduced rate of mitochondrial protein synthesis observed in lymphoblastoid cell lines (14). In mutant cell lines, a variable decrease in level of seven mtDNA-encoded polypeptides was observed in each protein. However, the levels of polypeptides in mutants relative to that in controls did not correlate with either the number or proportion of histidine codons, in contrast to what was previously shown in cells carrying the m.7445A>G mutation in the precursor of tRNA^Ser(UCN)^ (16) or the MERRF-associated 8344A>G mutation in tRNA^Lys^ gene (40). The improper aminoacylation may contribute to the variable decrease of each polypeptide in mutant cell lines. The impairment of mitochondrial translation then resulted in the respiration defects. In particular, there were very significant correlations between the rate of mitochondrial protein synthesis, and basal OCR, or ATP-linked OCR, maximal OCR in the control and mutant cell lines. This correlation is clearly consistent with the importance that a failure in tRNA^His^ metabolism plays a critical role in producing their respiration defects, as in the cases of cells carrying the deafness-associated tRNA^Ser(UCN)^ 7445A>G, 7472insC and 7511T>C mutations (16,18,41).

The respiratory deficiency caused by the tRNA^His^ 12201T>C mutation results in decreased efficiency of the mitochondrial ATP synthesis. In this study, 31% drop in mitochondrial ATP production in cybrid carrying the m.12201T>C mutation was much lower than those in cells carrying MERRF-associated tRNA^Lys^ 8344A>G and MELAS-associated tRNA^Leu(UUR)^ 3243A>G mutations (42,43). Alternatively, the reduction in mitochondrial ATP production in mutant cells was likely a consequence of the decrease in the proton electrochemical potential gradient of mutant mitochondria (42). As a result, cells carrying the mtDNA mutation may be particularly sensitive to increased ATP demand. Furthermore, the deficient activities of respiratory chain complexes caused by tRNA mutations often alter mitochondrial membrane potentials, which is a key indicator of cellular viability (44). Indeed, mitochondrial membrane potentials reflect the pumping of hydrogen ions across the inner membrane during the process of electron transport and oxidative phosphorylation (45). In this study, 80% reduction in mitochondrial membrane potential was observed in mutant cell lines carrying the m.12201T>C mutation. The defects in mitochondrial membrane potential may be due to strongly decreased efficiency of respiratory chain-mediated proto extrusion for the matrix, as in the case of other tRNA mutations (42,46). The impairment of both oxidative phoshorylation and mitochondrial membrane potential would elevate the production of ROS in mutant cells carrying the m.12201T>C mutation. The overproduction of ROS can establish a vicious cycle of oxidative stress in the mitochondria, thereby damaging mitochondrial and cellular proteins, lipids and nuclear acids (47). The hair cells and cochlear neurons may be preferentially involved because they are somehow exquisitely sensitive to subtle imbalance in cellular redox state or increased level of free radicals (48). This would lead to the dysfunction or death of cochlear and vestibular cells, thereby producing a phenotype of hearing loss.

In summary, our findings convincingly demonstrate the pathogenic mechanism leading to an impaired oxidative phosphorylation in cybrid cell lines carrying the deafness-associated tRNA^His^ 12201T>C mutation. The m.12201T>C mutation alters the secondary structure and function of tRNA. A failure in tRNA metabolism impaired mitochondrial translation and respiration. As a result, this respiratory deficiency reduced mitochondrial ATP production and membrane potentials. Consequently, the mutation leads to the increasing production of oxidative reactive species and subsequent hearing loss. Thus, our findings may provide the new insights into the understanding of pathophysiology of maternally inherited hearing loss.

## SUPPLEMENTARY DATA

Supplementary Data are available at NAR Online.

SUPPLEMENTARY DATA

## References

[1] Fischel-Ghodsian N. (1999). Mitochondrial deafness mutations reviewed. Hum. Mutat..

[2] Gutierrez Cortes N., Pertuiset C., Dumon E., Borlin M., Hebert-Chatelain E., Pierron D., Feldmann D., Jonard L., Marlin S., Letellier T. (2012). Novel mitochondrial DNA mutations responsible for maternally inherited nonsyndromic hearing loss. Hum. Mutat..

[3] Guan M.X. (2011). Mitochondrial 12S rRNA mutations associated with aminoglycoside ototoxicity. Mitochondrion.

[4] Prezant T.R., Agapian J.V., Bohlman M.C., Bu X., Oztas S., Qiu W.Q., Arnos K.S., Cortopassi G.A., Jaber L., Rotter J.I. (1993). Mitochondrial ribosomal RNA mutation associated with both antibiotic-induced and non-syndromic deafness. Nat. Genet..

[5] Zhao H., Li R., Wang Q., Yan Q., Deng J.H., Han D., Bai Y., Young W.Y., Guan M.X. (2004). Maternally inherited aminoglycoside-induced and nonsyndromic deafness is associated with the novel C1494T mutation in the mitochondrial 12S rRNA gene in a large Chinese family. Am. J. Hum. Genet..

[6] Zheng J., Ji Y., Guan M.X. (2012). Mitochondrial tRNA mutations associated with deafness. Mitochondrion.

[7] Ruiz-Pesini E., Lott M.T., Procaccio V., Poole J.C., Brandon M.C., Mishmar D., Yi C., Kreuziger J., Baldi P., Wallace D.C. (2007). An enhanced MITOMAP with a global mtDNA mutational phylogeny. Nucleic Acids Res..

[8] Goto Y., Nonaka I., Horai S. (1990). A mutation in the tRNA^Leu(UUR)^ gene associated with the MELAS subgroup of mitochondrial encephalomyopathies. Nature.

[9] Fischel-Ghodsian N., Prezant T.R., Fournier P., Stewart I.A., Maw M. (1995). Mitochondrial mutation associated with nonsyndromic deafness. Am. J. Otolaryngol..

[10] Tiranti V., Chariot P., Carella F., Toscano A., Soliveri P., Girlanda P., Carrara F., Fratta G.M., Reid F.M., Mariotti C. (1995). Maternally inherited hearing loss, ataxia and myoclonus associated with a novel point mutation in mitochondrial tRNA^Ser(UCN)^ gene. Hum. Mol. Genet..

[11] del Castillo F.J., Villamar M., Moreno-Pelayo M.A., Almela J.J., Morera C., Adiego I., Moreno F., del Castillo I. (2002). Maternally inherited non-syndromic hearing impairment in a Spanish family with the 7510T>C mutation in the mitochondrial tRNA^Ser(UCN)^ gene. J. Med. Genet..

[12] Sue C.M., Tanji K., Hadjigeorgiou G., Andreu A.L., Nishino I., Krishna S., Bruno C., Hirano M., Shanske S., Bonilla E. (1999). Maternally inherited hearing loss in a large kindred with a novel T7511C mutation in the mitochondrial DNA tRNA^Ser(UCN)^ gene. Neurology.

[13] Tang X., Li R., Zheng J., Cai Q., Zhang T., Gong S., Zheng W., He X., Zhu Y., Xue L. (2010). Maternally inherited hearing loss is associated with the novel mitochondrial tRNA^Ser(UCN)^ 7505T>C mutation in a Han Chinese family. Mol. Genet. Metab..

[14] Yan X., Wang X., Wang Z., Sun S., Chen G., He Y., Mo J.Q., Li R., Jiang P., Lin Q. (2011). Maternally transmitted late-onset non-syndromic deafness is associated with the novel heteroplasmic T12201C mutation in the mitochondrial tRNA^His^ gene. J. Med. Genet..

[15] Suzuki T., Nagao A., Suzuki T. (2011). Human mitochondrial tRNAs: biogenesis, function, structural aspects, and diseases. Annu. Rev. Genet..

[16] Guan M.X., Enriquez J.A., Fischel-Ghodsian N., Puranam R.S., Lin C.P., Maw M.A., Attardi G. (1998). The deafness-associated mitochondrial DNA mutation at position 7445, which affects tRNA^Ser(UCN)^ precursor processing, has long-range effects on NADH dehydrogenase subunit ND6 gene expression. Mol. Cell. Biol..

[17] Li Z., Liu Y., Yang L., Wang S., Guan M.X. (2008). Maternally inherited hypertension is associated with the mitochondrial tRNA^Ile^ A4295G mutation in a Chinese family. Biochem. Biophys. Res. Commun..

[18] Li X., Fischel-Ghodsian N., Schwartz F., Yan Q., Friedman R.A., Guan M.X. (2004). Biochemical characterization of the mitochondrial tRNA^Ser(UCN)^ T7511C mutation associated with nonsyndromic deafness. Nucleic Acids Res..

[19] King M.P., Attardi G. (1989). Human cells lacking mtDNA: repopulation with exogenous mitochondria by complementation. Science.

[20] King M.P., Attadi G. (1996). Mitochondria-mediated transformation of human rho(0) cells. Methods Enzymol..

[21] Guan M.X., Fischel-Ghodsian N., Attardi G. (2001). Nuclear background determines biochemical phenotype in the deafness-associated mitochondrial 12S rRNA mutation. Hum. Mol. Genet..

[22] Guan M.X., Fischel-Ghodsian N., Attardi G. (1996). Biochemical evidence for nuclear gene involvement in phenotype of non-syndromic deafness associated with mitochondrial 12S rRNA mutation. Hum. Mol. Genet..

[23] King M.P., Attardi G. (1993). Post-transcriptional regulation of the steady-state levels of mitochondrial tRNAs in HeLa cells. J. Biol. Chem..

[24] Jia Z., Wang X., Qin Y., Xue L., iang P, Meng Y., Shi S.Y., Mo JQ., Guan M.X (2013). Coronary heart disease is associated with a mutation in mitochondrial tRNA. Hum. Mol. Genet..

[25] Li R., Guan M.X. (2010). Human mitochondrial leucyl-tRNA synthetase corrects mitochondrial dysfunctions due to the tRNA^Leu(UUR)^ A3243G mutation, associated with mitochondrial encephalomyopathy, lactic acidosis, and stroke-like symptoms and diabetes. Mol. Cell. Biol..

[26] Enriquez J.A., Attardi G. (1996). Analysis of aminoacylation of human mitochondrial tRNAs. Methods Enzymol..

[27] Dranka B.P., Benavides G.A., Diers A.R., Giordano S., Zelickson B.R., Reily C., Zou L., Chatham J.C., Hill B.G., Zhang J. (2011). Assessing bioenergetic function in response to oxidative stress by metabolic profiling. Free Radic. Biol. Med..

[28] Schneider L., Giordano S., Zelickson B.R., M S.J., G A.B., Ouyang X., Fineberg N., Darley-Usmar V.M., Zhang J. (2011). Differentiation of SH-SY5Y cells to a neuronal phenotype changes cellular bioenergetics and the response to oxidative stress. Free Radic. Biol. Med..

[29] Zhou X., Qian Y., Zhang J., Tong Y., Jiang P., Liang M., Dai X., Zhou H., Zhao F., Ji Y. (2012). Leber's hereditary optic neuropathy is associated with the T3866C mutation in mitochondrial ND1 gene in three Han Chinese Families. Invest. Ophthalmol. Vis. Sci..

[30] Acton B.M., Jurisicova A., Jurisica I., Casper R.F. (2004). Alterations in mitochondrial membrane potential during preimplantation stages of mouse and human embryo development. Mol. Hum. Reprod..

[31] Trevelyan A.J., Kirby D.M., Smulders-Srinivasan T.K., Nooteboom M., Acin-Perez R., Enriquez J.A., Whittington M.A., Lightowlers R.N., Turnbull D.M. (2010). Mitochondrial DNA mutations affect calcium handling in differentiated neurons. Brain.

[32] Mahfouz R., Sharma R., Lackner J., Aziz N., Agarwal A. (2009). Evaluation of chemiluminescence and flow cytometry as tools in assessing production of hydrogen peroxide and superoxide anion in human spermatozoa. Fertil. Steril..

[33] Florentz C., Sohm B., Tryoen-Toth P., Putz J., Sissler M. (2003). Human mitochondrial tRNAs in health and disease. Cell. Mol. Life Sci..

[34] Hopper A.K., Phizicky E.M. (2003). tRNA transfers to the limelight. Genes Dev..

[35] Giege R., Sissler M., Florentz C. (1998). Universal rules and idiosyncratic features in tRNA identity. Nucleic Acids Res..

[36] Belostotsky R., Frishberg Y., Entelis N. (2012). Human mitochondrial tRNA quality control in health and disease: a channelling mechanism. RNA Biol..

[37] Kern A.D., Kondrashov F.A. (2004). Mechanisms and convergence of compensatory evolution in mammalian mitochondrial tRNAs. Nat. Genet..

[38] Hao R., Zhao M.W., Hao Z.X., Yao Y.N., Wang E.D. (2005). A T-stem slip in human mitochondrial tRNA^Leu(CUN)^ governs its charging capacity. Nucleic Acids Res..

[39] Chomyn A., Enriquez J.A., Micol V., Fernandez-Silva P., Attardi G. (2000). The mitochondrial myopathy, encephalopathy, lactic acidosis, and stroke-like episode syndrome-associated human mitochondrial tRNA^Leu(UUR)^ mutation causes aminoacylation deficiency and concomitant reduced association of mRNA with ribosomes. J. Biol. Chem..

[40] Enriquez J.A., Chomyn A., Attardi G. (1995). MtDNA mutation in MERRF syndrome causes defective aminoacylation of tRNA^Lys^ and premature translation termination. Nat. Genet..

[41] Toompuu M., Yasukawa T., Suzuki T., Hakkinen T., Spelbrink J.N., Watanabe K., Jacobs H.T. (2002). The 7472insC mitochondrial DNA mutation impairs the synthesis and extent of aminoacylation of tRNA^Ser(UCN)^ but not its structure or rate of turnover. J. Biol. Chem..

[42] James A.M., Sheard P.W., Wei Y.H., Murphy M.P. (1999). Decreased ATP synthesis is phenotypically expressed during increased energy demand in fibroblasts containing mitochondrial tRNA mutations. Eur. J. Biochem..

[43] Pallotti F., Baracca A., Hernandez-Rosa E., Walker W.F., Solaini G., Lenaz G., Melzi D'Eril G.V., Dimauro S., Schon E.A., Davidson M.M. (2004). Biochemical analysis of respiratory function in cybrid cell lines harbouring mitochondrial DNA mutations. Biochem. J..

[44] Szczepanowska J., Malinska D., Wieckowski M.R., Duszynski J. (2012). Effect of mtDNA point mutations on cellular bioenergetics. Biochim. Biophys. Acta.

[45] Chen Y.B., Aon M.A., Hsu Y.T., Soane L., Teng X., McCaffery J.M., Cheng W.C., Qi B., Li H., Alavian K.N. (2011). Bcl-xL regulates mitochondrial energetics by stabilizing the inner membrane potential. J. Cell Biol..

[46] de Andrade P.B., Rubi B., Frigerio F., van den Ouweland J.M., Maassen J.A., Maechler P. (2006). Diabetes-associated mitochondrial DNA mutation A3243G impairs cellular metabolic pathways necessary for beta cell function. Diabetologia.

[47] Bottger E.C., Schacht J. (2013). The mitochondrion: a perpetrator of acquired hearing loss. Hear. Res..

[48] Raimundo N., Song L., Shutt T.E., McKay S.E., Cotney J., Guan M.X., Gilliland T.C., Hohuan D., Santos-Sacchi J., Shadel G.S. (2012). Mitochondrial stress engages E2F1 apoptotic signaling to cause deafness. Cell.

