# Supplementary material for: A deafness-associated tRNAHis mutation alters the mitochondrial function, ROS production and membrane potential
Source: Nucleic Acids Res. 2014 Jun 11;42(12):8039–48. doi: 10.1093/nar/gku466 (PMC4081083; doi:10.1093/nar/gku466)
Supplement: SUPPLEMENTARY DATA [file supp_42_12_8039__index.html]

A deafness-associated tRNAHis mutation alters the mitochondrial function, ROS production and membrane potential — SUPPLEMENTARY DATA 

# A deafness-associated tRNAHis mutation alters the mitochondrial function, ROS production and membrane potential

## SUPPLEMENTARY DATA

**Files in this Data Supplement:**

- Supplementary Data
