# Supplementary material for: A deafness-associated tRNAHis mutation alters the mitochondrial function, ROS production and membrane potential
Source: Nucleic Acids Res. 2014 Jun 11;42(12):8039–48. doi: 10.1093/nar/gku466 (PMC4081083; doi:10.1093/nar/gku466)
Supplement: SUPPLEMENTARY DATA [file supp_gku466_nar-01074-a-2014-File010.pdf]

SUPPLEMENTAL DATA

1. Analysis for the m.12201T>C mutation in the tRNA^{His} gene in cybrid cell lines

To quantify the m.12201T>C mutation, the PCR segment (163 bp) was amplified using genomic DNA as the template and mismatched oligodeoxynucleotides [forward:5'-GGTGACACTATAGAATACTCAAGCTATGCATCAGACAACAGAGGCTTACGACCCCGTA -3' (nt 12169-12200); reverse: 5'-GGGGCCTAAGACCAATGGATAGCTG -3' (nt 12281-12305)] and subsequently digested with a restriction enzyme *CviQ1*. The m.12201T>C mutation together with the mismatched PCR primers created the site for this restriction enzyme. The 163 bp fragment cut by the enzyme *CviQ1* resulted in 105bp and 58bp fragments. Equal amounts of various digested samples were then analyzed by electrophoresis through 7% polyacrylamide gel. The proportions of digested and undigested PCR product were determined by the Image-Quant program after ethidium bromide staining to determine if m.12201T>C mutation is present in the homoplasmy in these subjects.

Supplemental Figure

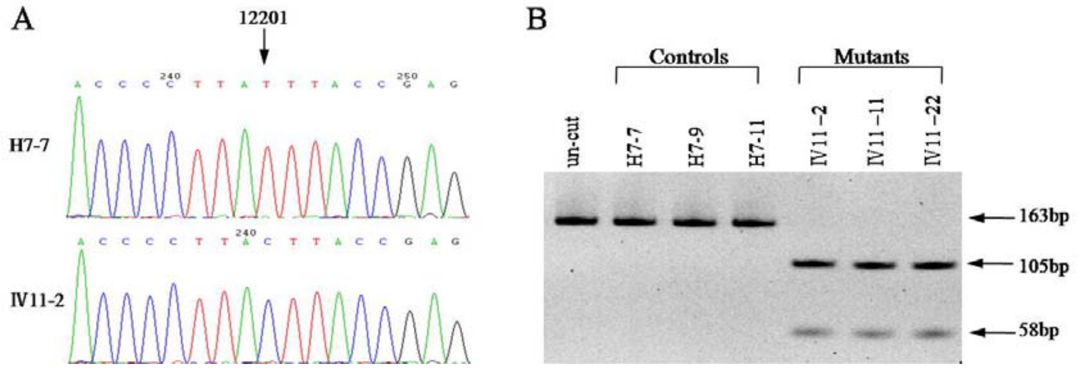

Figure S1. Identification and quantification of the m.12201T>C mutation in the tRNA^{His} gene. (A) Partial sequence eletropherograms of the tRNA^{His} gene from the cybrid cell lines of an affected individual (IV-11) and a Han Chinese control (H7), respectively. Arrows indicate the locations of the base changes at position 12201. (B) Quantification of the m.12201T>C mutation by PCR-RFLP. PCR products were digested with *CviQ1* and analyzed by electrophoresis in a 7% polyacrylamide gel stained with ethidium bromide. Patients and control cybrids are indicated.

Supplemental Table 1. mtDNA variants in one Han Chinese subject (IV-11) with deafness and one Han Chinese control subject (H7)

Gene	Position	Replacement	Conservation (H/B/M/X)[a]	CRS[b]	Control (H7)	Affected Subject (IV-11)	Previously Reported[c]
D-loop	73	A to G		A	G	G	Yes
	152	T to C		T	C	C	Yes
	249	delA		A	delA	delA	Yes
	263	A to G		A	G	G	Yes
	309	insC		C		insC	Yes
	310	T to CTC		T	CTC		Yes
	315	insC		C		insC	Yes
	460	T to C		T		C	Yes
	489	T to C		T	C	C	Yes
	16185	C to T		C	T	T	Yes
	16189	delT		T		delT	Yes
	16223	C to T		C	T	T	Yes
	16260	C to T		C	T	T	Yes
	16298	T to C		T	C	C	Yes
	16519	T to C		T	C	C	Yes
12S rRNA	709	G to A	G/A/A/-	G	A	A	Yes
	750	A to G	A/G/G/-	A	G	G	Yes
	1438	A to G	A/A/A/G	A	G	G	Yes
16S rRNA	2706	A to G	A/G/A/A	A	G	G	Yes
ND1	3396	T to C		T	C		Yes
	3405	A to G		A	G		Yes
ND2	4715	A to G		A	G	G	Yes
	4769	A to G		A	G	G	Yes
CO1	6752	A to G		A	G	G	Yes
	7028	C to T		C	T	T	Yes
	7196	C to A		C	A	A	Yes
	7250	A to G		A	G		Yes
CO2	7853	G to A (Val to Ala)	V/I/I/V	G	A		Yes
A6	8584	G to A (Ala to Thr)	A/V/V/I	G	A	A	Yes
	8701	A to G (Thr to Ala)	T/S/L/Q	A	G	G	Yes
	8860	A to G (Thr to Ala)	T/A/A/T	A	G	G	Yes
	9090	T to C		T	C	C	Yes
CO3	9540	T to C		T	C	C	Yes
ND3	10208	T to C		T	C	C	Yes
	10398	A to G (Thr to Ala)	T/T/T/A	A	G	G	Yes
	10400	C to T		C	T	T	Yes
ND4	10873	T to C		T	C	C	Yes
	11719	G to A		G	A	A	Yes
	11782	C to T		C	T	T	Yes

tRNA[His]	12201	T to C	T/T/T/T	T		C	Yes
ND5	12705	C to T		C	T	T	Yes
CYTB	14766	C to T(Thr to Ile)	T/S/I/S	C	T	T	Yes
	14783	T to C		T	C	C	Yes
	15043	G to A		G	A	A	Yes
	15301	G to A		G	A	A	Yes
	15326	A to G (Thr to Ala)	T/M/I/I	A	G	G	Yes
	15487	A to T		A	T	T	Yes
	15784	T to C		T	C	C	Yes

[a] Conservation of amino acid for polypeptides or of nucleotide for rRNAs in human (H), mouse (M), bovine (B), and *Xenopus laevis* (X)

[b] CRS, Cambridge reference sequence

[c] See online mitochondrial genome database: http//www.mitomap.org and http://www.genpat.uu.se/mtDB/

Supplemental Table 2. Usage of Histidine codons in human mitochondrial polypeptides and the average rate of the individual polypeptide in the mutant cell lines related to the average value in the control cell lines.

Gene	Number of Amino acids	Number of Histidine codons	Histone codons density (%)	Rate of synthesis of polypeptide in mutants (% of controls ± 2S.E.M.)
A6	227	6	2.6%	61±18
ND1	318	2	3.8%	34±18
ND4	459	13	2.8%	64±8
ND5	604	14	2.3%	60±19
CO1	514	18	3.5%	43±32
CO2	225	6	2.6%	19±7
CYTB	380	12	3.2%	63±28
